# Supplementary material for: Differential Requirement for IRGM Proteins during Tuberculosis Infection in Mice
Source: Infect Immun. 2023 Jan 11;91(2):e00510-22. doi: 10.1128/iai.00510-22 (PMC9933630; doi:10.1128/iai.00510-22)
Supplement: Supplemental file 1 — Fig. S1 to S3. Download iai.00510-22-s0002.pdf, PDF file, 2.3 MB [file iai.00510-22-s0002.pdf]

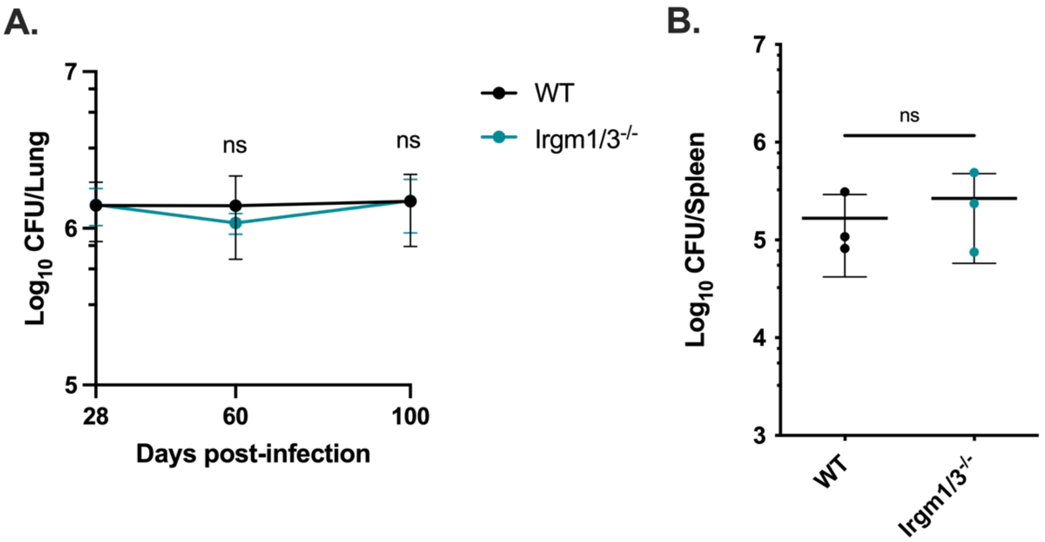

FIG S1. *Irgm1/3*$^{-/-}$ mouse TB phenotypes during long-term infection. Following aerosol infection with *Mtb* H37Rv (Day 0 dose of 50-150 CFUs), CFUs were quantified from the lungs (A) and spleens (B) of mice at 60- and 100-days post-infection (A) or 100 days post-infection (B). Each point represents a single mouse, data are from one experiment, with 3-4 female mice per group. Statistics were determined via Mann-Whitney test (ns = not significant).

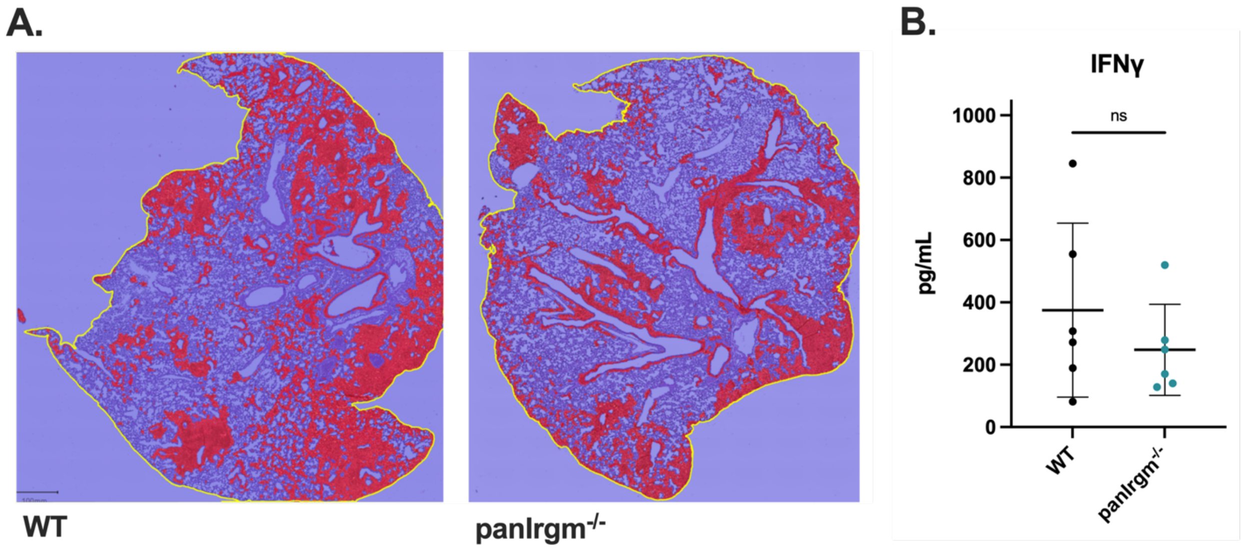

FIG S2. Lung damage quantification and phenotyping of pan*Irgm*$^{-/-}$ mice at 5 weeks post-infection (A) QuPath pixel classifier estimates of relative damaged area in representative H&E-stained lung sections from mice, collected at 5 weeks post-infection. (B) IFNγ cytokine concentration in lung homogenates from infected mice at 5 weeks post-infection. Statistics were determined via Mann-Whitney test (ns = not significant).

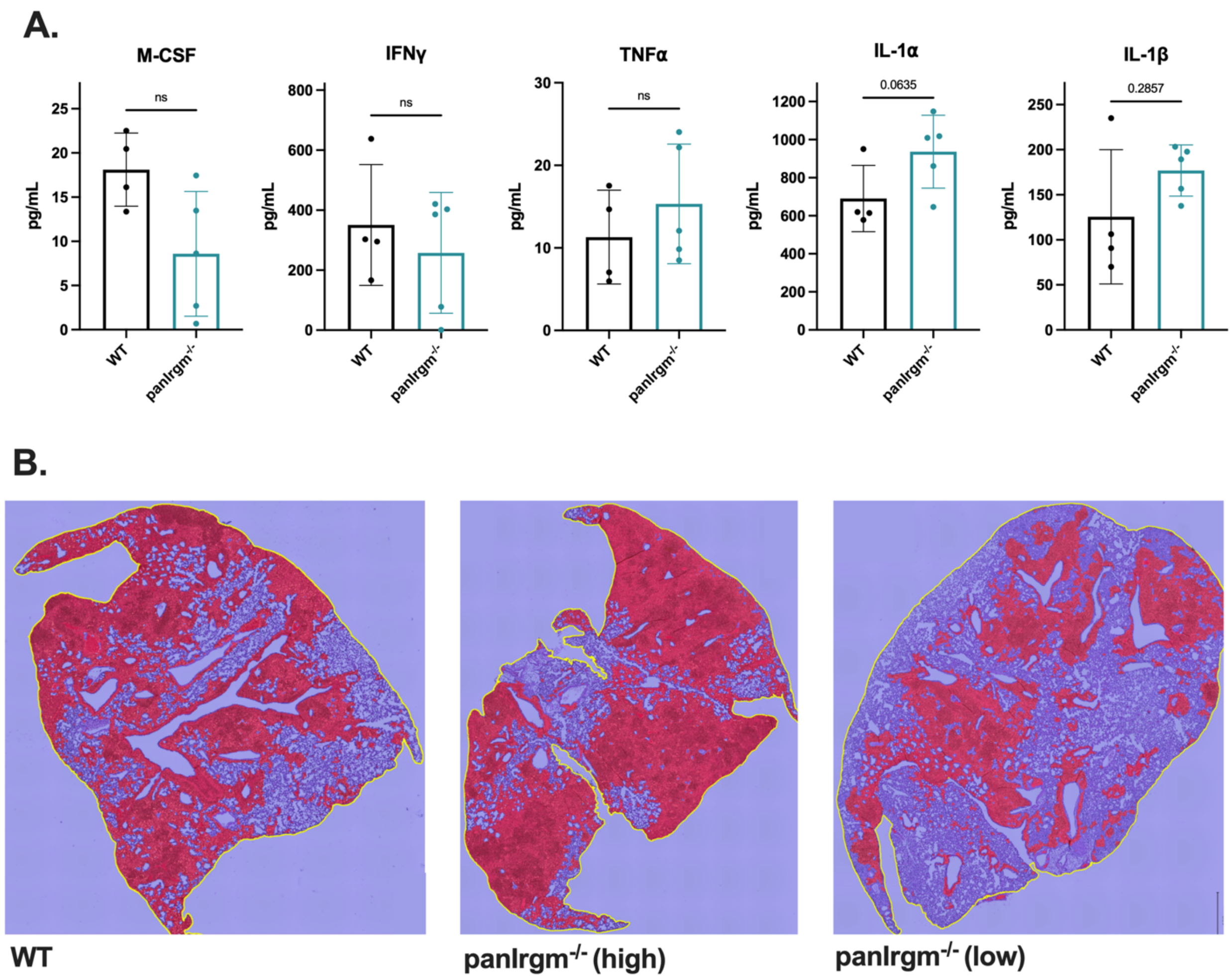

FIG S3. **Lung damage quantification and phenotyping of pan*Irgm*$^{-/-}$ mice at 24 weeks post-infection** (A) Concentration of cytokines in lung homogenates from infected female mice at 24 weeks post-infection. Statistics were determined by Mann-Whitney test (ns = not significant, or exact *P* value shown for trends above significance threshold). (B) QuPath pixel classifier estimates of relative damaged area in representative H&E-stained lung sections from mice, collected at 24 weeks post-infection.
